# Comparative Genomics of Cultured and Uncultured Strains Suggests Genes Essential for Free-Living Growth of *Liberibacter*

**DOI:** 10.1371/journal.pone.0084469

**Published:** 2014-01-08

**Authors:** Jennie R. Fagen, Michael T. Leonard, Connor M. McCullough, Janaka N. Edirisinghe, Christopher S. Henry, Michael J. Davis, Eric W. Triplett

**Affiliations:** 1 Microbiology and Cell Science Department, Institute of Food and Agricultural Sciences, University of Florida, Gainesville, Florida, United States of America; 2 Mathematics and Computer Science Division, Argonne National Laboratory, Argonne, Illinois, United States of America; 3 Plant Pathology Department, Citrus Research and Development Center, Institute of Food and Agricultural Sciences, University of Florida, Lake Alfred, Florida, United States of America; Hospital for Sick Children, Canada

## Abstract

The full genomes of two uncultured plant pathogenic *Liberibacter*, *Ca*. Liberibacter asiaticus and *Ca*. Liberibacter solanacearum, are publicly available. Recently, the larger genome of a closely related cultured strain, *Liberibacter crescens* BT-1, was described. To gain insights into our current inability to culture most *Liberibacter*, a comparative genomics analysis was done based on the RAST, KEGG, and manual annotations of these three organisms. In addition, pathogenicity genes were examined in all three bacteria. Key deficiencies were identified in *Ca*. L. asiaticus and *Ca*. L. solanacearum that might suggest why these organisms have not yet been cultured. Over 100 genes involved in amino acid and vitamin synthesis were annotated exclusively in *L. crescens* BT-1. However, none of these deficiencies are limiting in the rich media used to date. Other genes exclusive to *L. crescens* BT-1 include those involved in cell division, the stringent response regulatory pathway, and multiple two component regulatory systems. These results indicate that *L. crescens* is capable of growth under a much wider range of conditions than the uncultured *Liberibacter* strains. No outstanding differences were noted in pathogenicity-associated systems, suggesting that *L. crescens* BT-1 may be a plant pathogen on an as yet unidentified host.

## Introduction

The *Ca.* Liberibacter genus contains suspected plant pathogens as well as plant endophytes [1]. Collectively, *Ca*. L. asiaticus (CLas), *Ca*. L. africanus (CLaf), and *Ca*. L. americanus (CLam) cause a devastating disease on citrus known as Citrus Greening or Huanglongbing (HLB)[2], [3], [4]. *Ca*. L. solanacearum is the suspected causative agent of similar diseases on tomato and potato referred to as psyllid yellows and zebra chip (ZC), respectively [5], [6]. These bacteria remain uncultured in the laboratory, slowing progress toward description and the development of effective treatments. The closest cultured relative to the *Ca*. Liberibacter genus, *Liberibacter crescens*, and its genome were recently described [7]. A comparison of this genome to the uncultured *Liberibacter* is anticipated to broaden our understanding of the nature of genome reduction and its effect on culturability, as well as aid in the development of a *Liberibacter* culturing medium.

To date, three *Liberibacter* genomes have been sequenced including *Liberibacter crescens* BT-1, *Ca*. Liberibacter asiaticus psy62 (CLas), and *Ca*. Liberibacter solanacearum ZC1 (CLso). The genome sizes and other relevant characteristics of the strains from which they were derived are presented in Table 1. Genome comparisons have previously been published using CLas, CLso, and organisms of the same family, Rhizobiaceae [8], [9]. This investigation adds value by comparing a cultured relative of the *Liberibacter* genus, and is anticipated to provide further insight into the limitations on cultivating other *Liberibacter* species. The genome of *L. crescens* is approximately 1.50 Mb [7], while the reduced genomes of CLas and CLso are 1.23 Mb and 1.26 Mb, respectively [9], [10]. The genetic information lacking in CLas and CLso is potentially responsible for observed disparities in growth and virulence between *L. crescens* and these *Liberibacter* species.

**Table 1:** Description of *Liberibacter* species.

	*L. crescens* BT-1‡	*Ca*. L. asiaticus psy62^†^	*Ca*. L. solanacearum ZC1^◊^
Class	Alpha- proteobacteria	Alpha-proteobacteria	Alpha-proteobacteria
Cultured	Yes	No	No
Genome size	1.50 Mb	1.23 Mb	1.26 Mb
GC content	35.4%	35.2%	36.5%
Primary plant host	Babaco papaya	Citrus	Tomato, potato
Tissue colonized	Phloem	Phloem	Phloem
Disease caused	Unknown	Huanglongbing	Zebra chip/psyllid yellows
Insect vector	Unknown	*D. citri*	*B. cockerelli*

A brief comparison of the three sequenced *Liberibacter* species. ‡ Leonard et al 2012; † Duan et al 2009; ◊Lin et al 2011.

## Materials and Methods

### Culturing liberibacter crescens

*L. crescens* is grown on the artificial medium BM7 prepared as follows: 550 ml water, 2 g alpha-ketogluterate, 10 g ACES buffer, 3.75 g KOH, adjust pH to 6.9 and sterilize in the autoclave for 15 minutes at 121°C. After cooling, 150 mL of fetal bovine serum (Hyclone) and 300 mL of TMN-FH (Hyclone) was added. *L. crescens* cultures were grown at 28°C in a shaking incubator at 125 rpm. DNA extraction, genomic sequencing, and microscopy of the strain were described previously [7].

### Genome comparisons

The genomes of *Liberibacter crescens* [CP003789.1], *Ca*. Liberibacter asiaticus [NC_012985.3], and *Ca.* Liberibacter solanacearum [NC_014774.1] were analyzed. All genomes were re-annotated using the RAST pipeline [11], although CLas and CLso, were already present in the SEED database. The initial annotation and subsystem assignments were done in RAST and accessed with the SEED viewer [12]. The total features in each subsystem were determined from SEED v. nmpdr_29 on biologin04.mcs.anl.gov and the percentage of each subsystem covered by the genomes was calculated (Table 2). Disparate functions were noted in the SEED viewer and investigated in KEGG [13], [14]. All metabolically pertinent functional losses were confirmed manually using a BLAST search of the target genome [15].

**Table 2:** Genomic coverage of SEED subsystems.

Subsystem	Features in Subsystem	*L. crescens* genes (%)	*Ca.* L. asiaticus genes (%)	*Ca*. L. solanacearum genes (%)	*B. bacilliformis* KC583 genes (%)	*X. fastidiosa* 9a5C genes (%)
Amino Acids and Derivatives	863	154 (17.8)	69 (8.0)	67 (7.8)	104 (12.1)	203 (23.5)
Carbohydrates	1859	56 (3.0)	41 (2.2)	41 (2.2)	51 (2.7)	81 (4.4)
Cell Division and Cell Cycle	111	14 (12.6)	13 (11.7)	12 (10.8)	23 (20.7)	33 (29.7)
Cell Wall and Capsule	687	70 (10.2)	65 (9.5)	63 (9.2)	67 (9.8)	128 (18.6)
Cofactors, Vitamins, Prosthetic Groups Pigments	776	86 (11.1)	69 (8.9)	72 (9.3)	94 (12.1)	162 (20.9)
DNA Metabolism	311	81 (26.0)	82 (26.4)	80 (25.7)	73 (23.5)	131 (42.1)
Dormancy and Sporulation	205	1 (0.5)	1 (0.5)	1 (0.5)	1 (0.5)	1 (0.5)
Fatty Acids, Lipids, and Isoprenoids	270	39 (14.4)	28 (10.4)	28 (10.4)	35 (13.0)	76 (28.1)
Membrane Transport	379	26 (6.9)	18 (4.7)	18 (4.7)	31 (8.2)	84 (22.2)
Metabolism of Aromatic Compounds	440	5 (1.1)	2 (0.5)	2 (0.5)	3 (0.7)	1 (0.2)
Miscellaneous	59	28 (47.5)	28 (47.5)	23 (39.0)	37 (62.7)	17 (28.8)
Motility and Chemotaxis	210	39 (18.6)	14 (6.7)	15 (7.1)	5 (2.4)	0 (0.0)
Nucleosides and Nucleotides	240	45 (18.8)	52 (21.7)	47 (19.6)	52 (21.7)	62 (25.8)
Phosphorus Metabolism	82	18 (22.0)	11 (13.4)	11 (13.4)	13 (15.9)	28 (34.1)
Potassium metabolism	52	4 (7.7)	3 (5.8)	3 (5.8)	7 (13.5)	7 (13.5)
Protein Metabolism	808	140 (17.3)	135 (16.7)	106 (13.1)	188 (23.3)	215 (26.6)
Regulation and Cell Signaling	333	18 (5.4)	0 (0.0)	0 (0.0)	4 (1.2)	51 (15.3)
Respiration	902	42 (4.7)	44 (4.9)	41 (4.5)	58 (6.4)	73 (8.1)
RNA metabolism	659	91 (13.8)	90 (13.7)	92 (14.0)	105 (15.9)	115 (17.5)
Secondary Metabolism	172	4 (2.3)	0 (0.0)	0 (0.0)	0 (0.0)	8 (4.7)
Stress Response	457	40 (8.8)	28 (6.1)	26 (5.7)	42 (9.2)	66 (14.4)
Sulfur Metabolism	147	2 (1.4)	2 (1.4)	2 (1.4)	2 (1.4)	20 (13.6)
Virulence, Disease and Defense	1553	28 (1.8)	19 (1.2)	20 (1.3)	32 (2.1)	37 (2.4)

The total number of genes annotated in each SEED subsystem is shown. The number of subsystem genes identified in each studied genome is shown in bold, with the percent-coverage in parenthesis. Additional manual curation, independent of RAST, is not reflected in this table.

### Virulence effectors

Previously identified secreted proteins from *Ca.* L. asiaticus psy62 [16] were checked bidirectionally against the *L. crescens* and *Ca.* L. solanacearum genomes using BLAST. Unidirectional hits and those with an e-value of greater than 0.001 were considered to be absent in the queried genome.

### Prophage comparisons

Taxonomic prediction of prophage regions was performed by Phage RAST [11]. Coding sequence prediction was also performed by Phage RAST through their self-trained version of GeneMark [17]. Functional prediction of coding sequences was performed by the RAST pipeline and a BLAST search against the EBI phage database and NCBI non-redundant (nr) database. Sequence comparison between *Ca*. L. asiaticus, *Ca*. L. solanacearum, and *L. crescens* was performed by the RAST pipeline and a BLAST search was conducted against databases of coding sequences for each prophage.

## Results and Discussion

### Liberibacter crescens cultural characteristics

*L. crescens* culture has a doubling time of 36.7 hours and reaches a maximum optical density of approximately 0.57 after 100 hours of incubation in liquid BM7 medium at 28°C (Figure S1). Circular, cream-colored colonies begin to appear 8–10 days after inoculation on solid BM7.

### Genome comparison

**Figure 1:**
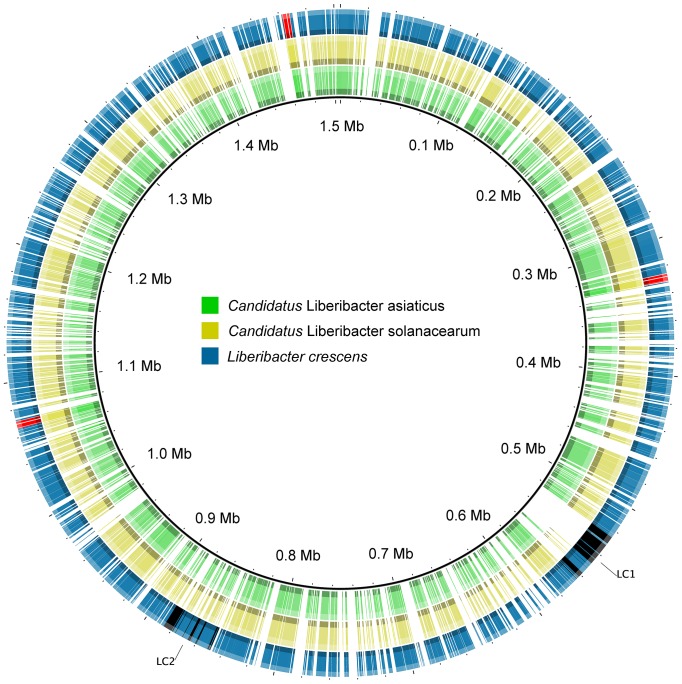
*Liberibacter* genome alignment. Microsynteny was observed across all three sequenced *Liberibacter* genomes, using *L. crescens* as a reference. From outer to inner circle: *L. crescens* (blue), CLso (yellow), CLas (green). Additionally, prophage regions (black) and rRNA operons (red) are denoted for *L. crescens*.

**Figure 2:**
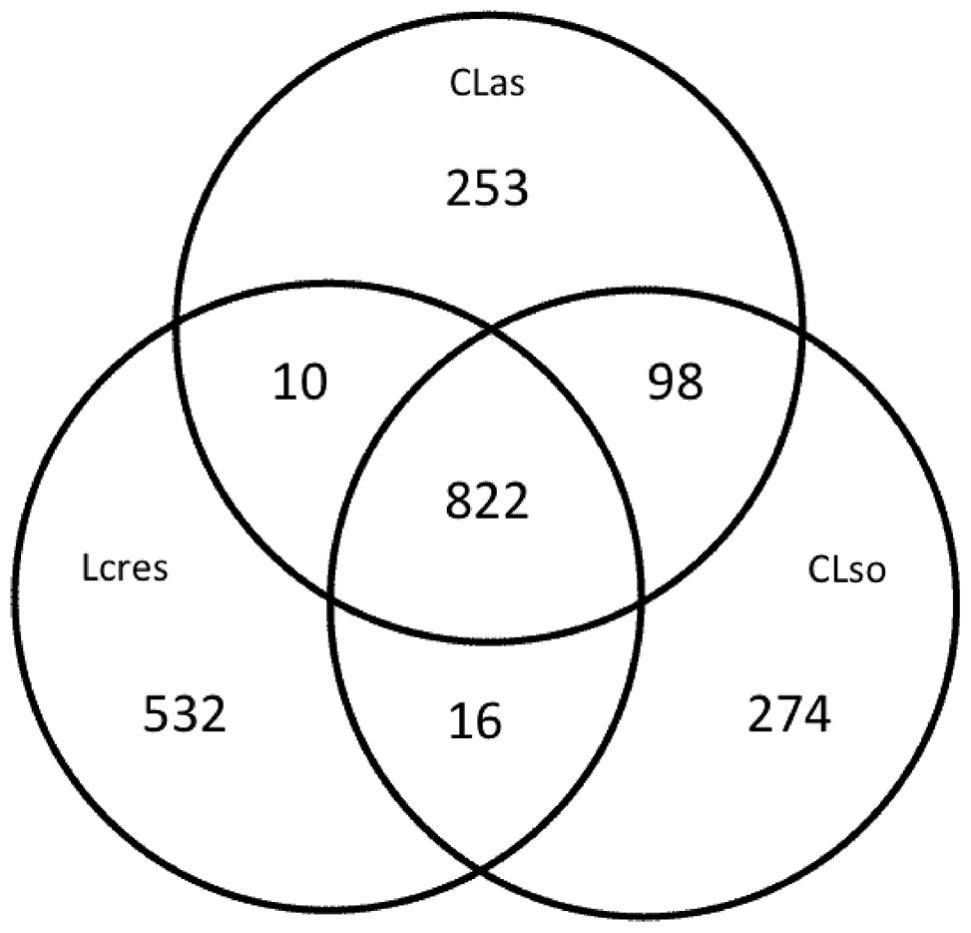
Venn Diagram showing genes shared between all three *Liberibacter* genomes. A total of 2005 separate genes were identified based on RAST orf predictions. A sequence-based comparison showed that 822 of these are shared between all *Liberibacter* species studied. *Liberibacter crescens* (Lcres); *Ca.* Liberibacter asiaticus (CLas); and *Ca*. Liberibacter solanacearum (CLso).

An alignment of all three *Liberibacter* genomes revealed no large continuous regions to be unique to *L. crescens* (Figure 1). This demonstrates that the approximately 0.25 Mb of additional sequence in the *L. crescens* genome is not the result of the incorporation of a large segment of DNA or the loss of a similar segment in the CLas and CLso genomes. Rather it seems that genome reduction in CLas and CLso has taken place uniformly across their genomes. Initial annotations of the three *Liberibacter* genomes share 697 gene functions. A total of 207, 9, and 13 functions were unique to *L. crescens*, *Ca.* L. asiaticus, and *Ca.* L. solanacearum, respectively (Table 2, Figure 2, Figure S2). A nucleotide sequence based comparison of the *Liberibacter* genomes showed 235 hypothetical genes of unknown function to be present in *L. crescens* but not CLas or CLso (Table S1).

### Biosynthesis

#### Amino Acids

Biosynthetic pathways and transporters for amino acids were examined in the three genomes. In total, the uncultured *Liberibacter* species lack the ability to synthesize six amino acids that are produced by *L. crescens* (Table 3). De novo synthetic pathways for proline, phenylalanine, tryptophan, cysteine, tyrosine, and histidine are found in *L. crescens* genome but are not produced by CLas and CLso. This result is consistent with findings in previous *Liberibacter* genomic analyses [7], [9], [10]. *Liberibacter crescens* has a complete shikimic acid pathway, which contributes to the novo synthesis of the aromatic amino acids tyrosine, phenylalanine, and tryptophan. The shikimic acid pathway is absent in CLas and CLso (Figure S3). However, multiple specific and nonspecific amino acid transporters are encoded by all *Liberibacter* species (Figure S2), which are expected to import essential amino acids or precursors from the plant or insect host. These differences in amino acid biosynthesis are not expected to impact culturing efforts so long as a complete set of amino acids and precursors are available in the growth medium.

**Table 3:** Comparison of amino acid biosynthesis and transport in the *Liberibacter* species.

	*L. crescens* BT-1		*Ca.* L. asiaticus		*Ca.* L. solanacearum	
Amino Acid	Synthesis	Specific Transporter	Synthesis	Specific Transporter	Synthesis	Specific Transporter
Alanine	-	-	-	-	-	-
Valine	-	-	-	-	-	-
Leucine	-	-	-	-	-	-
Isoleucine	-	-	-	-	-	-
Proline	+	+	-	+	-	+
Methionine	-	+	-	-	-	-
Phenylalanine	+	-	-	-	-	-
Tryptophan	+	-	-	-	-	-
Glycine	+	+	+	+	+	+
Serine	+	-	+	-	+	-
Threonine	+	-	+	-	+	-
Cysteine	+	-	-	-	-	-
Asparagine	-	-	-	-	-	-
Glutamine	+	-	+	-	+	-
Tyrosine	+	-	-	-	-	-
Aspartic Acid	-	+	-	+	-	+
Glutamic Acid	-	-	-	-	-	-
Lysine	+	-	+	-	+	-
Arginine	+	-	+	-	+	-
Histidine	+	-	-	-	-	-
Total	12/20		6/20		6/20	

A general amino acid transporter was present in all three genomes and only additional, specific transporters are represented in this table.

#### Cofactors

**Figure 3:**
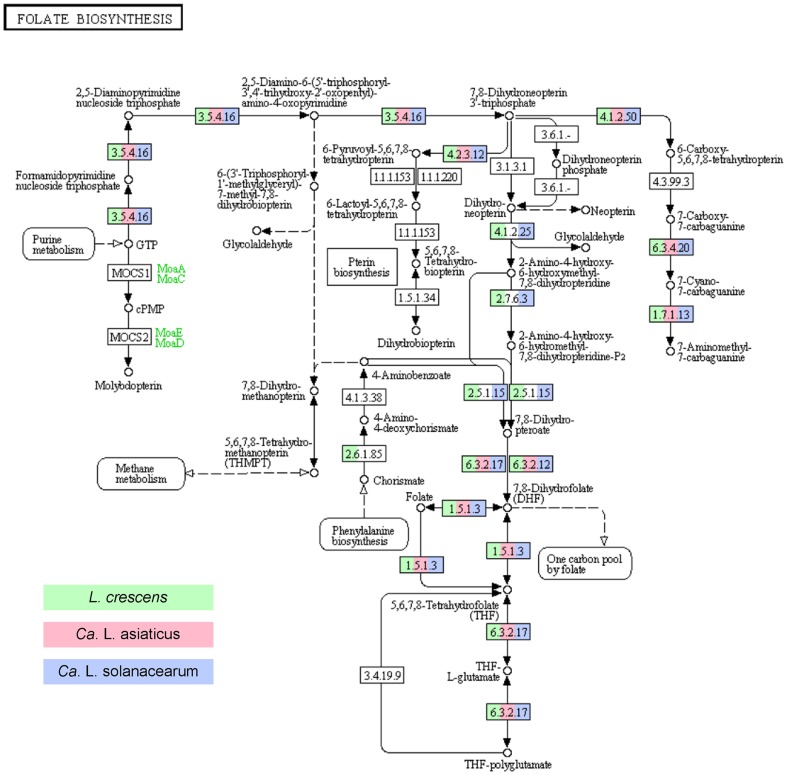
KEGG pathway indicating the extent of Folate Biosynthesis in *Liberibacter* species. All genomes lack alkaline phosphatase (EC 3.1.3.1.). This pathway is further incomplete in Ca. Liberibacter asiaticus, which has lost several enzymes for the production of Dihydrofolate (DHF).

Biosynthetic pathways of biotin, riboflavin, and pyridoxine were complete in all three genomes. Only *L. crescens* can produce (R)-pantothenate (Figure S4). CLas and CLso have a specific thiamine transporter not found in *L. crescens*; but no pantothenate transporter was identified (Table 4). The folate (vitamin B9) production pathway in all three *Liberibacter* genomes lacks a key alkaline phosphatase (EC 3.1.3.1). This enzyme is present in *Rhizobiaceae* species. The remainder of the pathway is present in *L. crescens* and CLso, both of which may be able to bypass the missing alkaline phosphatase and produce folate through the use of a multifunctional phosphatase. However, CLas lacks genes fol1, folB, folK, and folP and therefore the folate synthesis pathway is presumed to be nonfunctional (Figure 3). All *Liberibacter* species lack biosynthetic pathways for cobalamin, pyridoxal phosphate, and niacin. However, *L. crescens* has a niacin/H+ symporter (NiaP) not found in the other two *Liberibacter* genomes. NiaP in *Ralstonia solanacearum* transports nicotinate and is energy dependent [18]. The *L. crescens* NiaP sequence is most similar to those in *A. tumefaciens* and other *Rhizobiaceae*. All *Liberibacters* are able to recycle NAD, however, none of them are able to synthesize NAD from aspartate or tryptophan. Therefore, CLas and CLso must be importing nicotinate or NAD through an unidentified mechanism. This niacin transport requirement may contribute to the inability of CLas and CLso to grow in current media formulations.

**Table 4:** Comparison of cofactor de novo synthesis and transport in the *Liberibacter* species.

	*L. crescens* BT-1		*Ca.* L. asiaticus		*Ca.* L. solanacearum	
Cofactor	Synthesis	Specific Transporter	Synthesis	Specific Transporter	Synthesis	Specific Transporter
Thiamine (B1)	+	-	-	+	-	+
Riboflavin (B2)	+	-	+	-	+	-
Pyridoxine(B6)	+	-	+	-	+	-
Nicotinamide	-	-	-	-	-	-
Pantothenate (B5)	-*	-	-	-	-	-
Biotin (B7)	+	-	+	-	+	-
Folate (B9)	+	-	-	-	+	-
Cobalamin(B12)	-	-	-	-	-	-
pyridoxal phosphate (B6)	-	-	-	-	-	-
Niacin (B3)	-	+	-	-	-	-

*L. crescens is able to convert L-aspartate to panthothenate.

#### Polyamines

Putrescine and its derivatives, spermidine and norspermidine, are referred to collectively as polyamines. Polyamines are present in all branches of life and are thought to be a core metabolic requirement [19]. The putrescine production pathway is present in *L. crescens* but incomplete in CLas and CLso, which lack ornithine decarboxylase [EC:4.1.1.17] (Figure S5). CLas and CLso are able to metabolize putrescine but lack the necessary enzymes for de novo synthesis. The lack of polyamine synthesis in CLas and CLso may constitute an important host-dependence, which hinders culturing efforts. When the spermidine biosynthetic pathway is partially inhibited in *R. leguminosarum*, a reduced growth rate is observed [20]. *R. leguminosarum* possesses multiple polyamine transporters [21] and the growth of the spermidine synthesis mutant can be recovered with exogenous applications of spermidine, homospermidine, and norspermidine. The addition of these polyamines to the CLas/CLso growth medium may improve growth. However, no polyamine transporter that would import putrescine or its precursors was identified in CLas or CLso in this study or a previous in-depth prediction of ABC transporters in CLas [22].

### Central carbon metabolism

CLas and CLso were previously reported to lack the glucose-6-phosphate isomerase [9], [10] and this absence was confirmed here. Glucose-6-phosphate isomerase is present in *L. crescens*, which has a complete Embden-Meyerhof glycolytic pathway. CLas and CLso may circumvent the step catalyzed by glucose-6-phosphate isomerase by utilizing certain portions of the pentose phosphate pathway (PPP) (Figure S6). In such a scenario, two additional NADPH will be produced per glucose-1-phosphate in CLas and CLso.

### Electron transport/respiration

All three *Liberibacter* genomes encode NADH dehydrogenase (NuoA-N), succinate dehydrogenase (SdhA-D), an O type terminal respiratory oxidase (CyoABCDE), and an F-type ATPase. In addition to these, *L. crescens* has a cytochrome d type ubiquinol oxidase that was not detected in either CLas or CLso (Figure S2). The CydABCD of *L. crescens* has a close homologue in other members of the *Rhizobiacae* and therefore was most likely lost by CLas and CLso during genome reduction rather than acquired by *L. crescens* after the lineages diverged.

Cytochrome bd is unrelated to heme-copper oxidases and does not pump protons directly [23]. Cytochrome bd has a high O_2_ affinity and is often expressed under microaerobic or Fe-limited conditions whereas cytochrome o oxidase expression is often limited to oxygen-rich environments [23], [24]. CydABCD was found in other intracellular pathogens such as *Mycobacterium tuberculosis* F11 and *Leifsonia xyli* xyli CTCB07. However, based on the exclusive presence of Cyo in CLas and CLso, it is recommended that lowered O_2_ levels be avoided in culture. This is in stark contrast to the phloem environment that is characterized by greatly reduced O_2_ levels [25].

### Membrane transport

#### ABC Transporters

Transporters are of particular importance in bacteria that have a greatly reduced synthetic metabolism such as *Liberibacter* species. Thirteen ABC transporters were found in the *L. crescens* genome. This work pays particular attention to transporters present in *L. crescens* that were not identified in CLas or CLso. Of these thirteen ABC transporters, five were identified only in the *L. crescens* genome: *nia*P, *fts*EX, *fhu*BCD, *opp*ABCD, and *rax*B. These transporters may be unnecessary in the intracellular environment but still beneficial in culture.

The CLas and CLso genomes lack *fts*E and *fts*X, which are involved in cell division [26]. An *fts*E(Ts) mutation in *E. coli* results in deficient incorporation of K+-pump proteins (KdpA, Kup, TrkH, PhoA) into the cytoplasmic membrane [27]. Of these K+-pump genes, CLas and CLso have only *kup*. Kup is a low affinty K+ importer and in *E. coli* it functions during hyper-osmotic stress and in acidic environments similar to those of the phloem [28]. A large exogenous source of K+ ions may be necessary for proper growth and division of CLas and CLso.

CLas and CLso lack FhuBCD, which is responsible for the uptake or Fe3+ hydroxamate compounds such as ferrichrome [29]. Without this operon, CLas and CLso may have limited iron scavenging and intracellular iron regulation capabilities. CLso contains a high affinity Fe2+ transporter (EfeUOB). EfeU sequence from CLso has 54.4% amino acid sequence similarity to an iron permease protein in *L. crescens*; however, no homolog was identified in CLas. The EfeUOB transporter is active under iron limitation and low pH [30]. The presence of this system in CLso was previously reported [8], and the function in *L. crescens* is undetermined. All three *Liberibacter* genomes contain an Fe(II)/Manganese transporter (SitABCD) and the zinc transporter (ZnuABC). SitABCD is associated with virulence and resistance to oxidative stress [31].

The Opp ABC oligopeptide transporter is found only in *L. crescens*. OppABC functions in the recycling of cell wall peptides [32]. The operon has a different order in *L. crescens* (OppDABC) than in other organisms (OppABCD). The structure and sequence of the *L. crescens* OppDABC operon is most similar to that of the alphaproteobacteria *Roseobacter denitrificans* OCh114. Mutants of *Sinorhizobium meliloti* in oppA and oppB exhibit impaired growth on minimal medium supplemented with tetra peptides [33]. In *E. coli* Opp mutants lyse more quickly than wild type cells once all diaminopimelic acid (DAP) in the growth medium is exhausted. A consistent source of cell wall components in the intracellular environment may compensate for the lack of the system in CLas and CLso as well.

#### Phosphotransferase systems

No complete phosphotransferase systems (PTS) were identified in the studied *Liberibacter* genomes as previously noted by Lin et al 2011. *L. crescens* encodes proteins that participate in phosphtranferase systems (PTS) for mannose, ascorbate and nitrogen. Neither of the permease subunits of the mannose PTS were present in *L. crescens*. However, the cytoplasmic subunit, ManX, was identified. This is a pattern common to many alphaproteobacteria and the protein may serve a regulatory function [34]. *L. crescens* also encodes the EI (PtsP) and HPr (PtsO) of the nitrogen PTS which are not found in CLas or CLso. The nitrogen EIIA protein (PtsN) was not found in *L. crescens*.

The transmembrane component of the ascorbate PTS was present in all three *Liberibacter* genomes, but all genomes lacked the cytoplasmic components of this system. It is unclear if the transmembrane component is sufficient for the transport of ascorbate by *L. crescens*. However, all three *Liberibacter* species are assumed to utilize glucose as their primary carbon source.

#### Secretion Systems

All *Liberibacter* species have a complete Sec system, however, the twin arginine transport (TAT) system was only found in *L. crescens*. TAT has been shown to be important for the export of some virulence factors through the type 2 secretion system (T2SS). However, the T2SS was absent in all *Liberibacter* species studied. Type 1 secretion systems (T1SS) have been previously reported in CLas and CLsol [9], [10]. However, the tolC component was not identified in any of the *Liberibacter* genomes in this work. No other components of bacterial secretion systems were found in these organisms.

#### Other Transporters

The only transporter present in CLas and CLso, but absent in *L. crescens*, is an NttA-family ATP/ADP transporter. This protein is thought to allow CLas and CLso to utilize host-derived ATP and ADP [35]. No evidence was found in this genomic analysis to suggest that CLas and CLso are entirely dependent on an extracellular source of ATP/ADP.

### Regulation

#### Two Component Systems

There are four, two component signal transduction systems (TCS) present in *L. crescens* that are absent or incomplete in CLas and CLso: EnvZ/OmpR, DivJK, NtrXY, and ChvGI. This gives *L. crescens* an increased ability to regulate gene expression in response to extracellular stimuli.

EnvZ/OmpR regulates gene expression in response to changing osmotic pressure [36]. The absence of this system in CLas and CLso suggests that these two pathogens are sensitive to changes in osmotic pressure, which may occur during culturing. However, another cultured plant pathogen, *X. fastidiosa*, also lacks this system suggesting that the transition from an intracellular environment to growth in vitro does not required EnvZ/OmpR.

*L. crescens* has the histidine kinase, DivJ, which is not present in the CLas or CLso genomes. However, DivK, which is phosphorylated by DivJ, is present in both CLas and CLso. DivJ participates in cell division [37]. A DivJ null mutant in *Caulobacter crescentus* exhibited slowed growth and an altered phenotype; cells became slightly elongated with aberrant polar localization [38]. The absence of DivJ in CLas and CLso may be partly responsible for observed slow growth rates *in planta* but is unlikely to selectively hinder growth *in vitro*.

The NtrY/NtrX two component regulatory system seems to be degraded in CLas and CLso. An *ntrX*-like gene was found in CLso but an *ntrY* homologue was not identified. Neither *ntrX* nor *ntrY* were present in CLas. NtrY/NtrX was first annotated in *Axorhizobium caulinodans* PRS571 where it is involved in nitrogen fixation [39]. A knock out of *ntrX* in *A. caulinodans* lead to diminished growth on nitrate medium. This two component regulatory system may be important to nitrogen assimilation by *L. crescens* and allow it to grow more vigorously in culture.

*Liberibacter crescens* possesses the Chv regulatory gene cluster, ChvG/ChvI, similar to those found in *R. leguminosarum, S. meliloti,* and *A. tumefaciens*. ChvG responds to acidic conditions during plant wounding and activates ChvI, which regulates the expression of a broad range of virulence and metabolic genes [40]. A ChvI null mutation in *S. meliloti* 1021 exhibited decreased growth under acidic conditions, no extracellular polysacharide (EPS) production, reduced poly-3-hydroxybutyrate (PHB), and no growth on complex media [41]. Other studied alphaproteobacterial ChvG/ChvI proteins are required for host invasion and tumor formation [42]. Because of its association with growth on complex media, the lack of ChvG/ChvI in CLas and CLso provides valuable insight into the inability to cultivate these species.

CLas and CLso lack several sensor histidine kinases found in *L. crescens*. However, the CLas and CLso genomes share a sensory histidine kinase of unknown function that has no close homolog in *L. crescens*. This gene is feature YP_004062632.1 in *Ca*. L. solanacearum and YP_003064826.1 in *Ca.* L. asiaticus. The target of this histidine kinase has yet to be determined and its environmental trigger is unknown.

#### Copper Homeostasis

CLas and CLso lack multicopper oxidase (MCO) found in *L. crescens*. The multicopper oxidase in *L. crescens* is most closely related to the MCO in *Rhizobium leguminosarum* bv. viciae 3841. MCO is flanked by the same genes in *L. crescens* and *R. leguminosarum*. MCO has been implicated in copper tolerance and copper homeostasis, as well as oxidative stress response [43]. The *L. crescens* MCO is not thought to contribute directly to growth in culture.

#### Zinc Regulation

The zinc uptake regulation protein ZUR is present in *L. crescens* but is not found in CLas or CLso. ZUR regulates zinc uptake via the Znu ABC transporter [44]. Despite the missing regulator in CLas and CLso, the ZnuABC transporter is present in all three sequenced *Liberibacter* genomes. CLas and CLso possess the zinc transport mechanism, but are unable to regulate this uptake in the same manner as *L. crescens*.

#### Stringent Response

The stringent response gene, *spo*T, via (p)ppGpp synthetase II [45] is only found in *L. crescens*. In *Salmonella* infections, ppGpp mediates the rapid metabolic shift as the pathogen moves between environments. [46], [47]. SpoT and the surrounding gene cluster in *L. crescens* are closely related to those in *R. leguminosarum, A. tumefaciens,* and *S. meliloti 1021*. This system is therefore presumed to be functional. It seems unusual that intracellular pathogens such as CLas and CLso would not utilize the ppGpp system to mediate the switch from the insect vector to the plant phloem. This lack of widespread gene regulation may also be contributing to difficulties with maintaining these strains in culture.

The C4-type zinc finger protein family DksA/TraR is found in *L. crescens* but is not present in CLas or CLso. This zinc finger has been linked to ppGpp and the stringent response. DksA acts to overcome stalling of replication forks due to the global changes in transcription initiated by the stringent response [48]. CLas and CLso lack the stringent response, and are not expected to experience the associated, slowed chromosome replication. Therefore, DksA is likely unnecessary in CLas and CLso.

### Cell structure

#### Lipid A Biosynthesis

All *Liberibacter* species are predicted to produce lipid A, a subunit of lipopolysaccharide. However, only *L. crescens* possesses the membrane protein LpxL. An *lpxL* E. coli mutation is lethal at temperatures above 32.5 degrees C [49]. The absence of LpxL in CLas may explain recent observations in which controlled heating of CLas-infected citrus eliminates HLB infection under greenhouse conditions [50].

#### Peptioglycan Recycling

The peptidoglycan amino acid recycling system (*ami*D, *ldc*A, *ami*A, MCPase, OppD, MltB, slt) is present in *L. crescens* but absent in CLas and CLso (Table 5). Homologues for two other genes involved in cell wall recycling, *amp*G and *nag*Z, were absent in all *Liberibacter* species. An *ldc*A *E. coli* mutant spontaneously lyses at stationary phase [51]. Furthermore, the murein hydrolases MCPase, *ami*D, and *slt* are found only in the *L. crescens* genome. Murein hydrolases are involved in peptidoglycan recycling [52]. AmiD is a periplasmic N-acetylmyramoyl-L-alanine amidase reliant on the TAT pathway [53]. AmiD is the functional equivalent of AmpD, which is a cytoplasmic anhydro-N-acetylmuramyl-L-alanine amidase in *Escherichia coli* [54]. Two amiD copies are present in *L. crescens* with one copy in the prophage region. The murein hydrolases MltB and DacD are present in all *Liberibacter* genomes. The absence of cell wall recycling pathway components in the CLas and CLso genomes may contribute to the lack of sustained growth in culture, although this pathway is apparently not needed for CLas survival in plant or insect hosts.

**Table 5:** Peptidoglycan recycling systems of *Liberibacter* species.

Gene	Presence	Activity
amiD (ampD, oppC)	Lc*	anhydro-N-acetylmuramyl-L-alanine amidase
ldcA	Lc	Muramoyltetrapeptide carboxypeptidase
amiA	Lc	N-acetylmyramoyl-L-alanine amidase
MCPase	Lc	Muramoyltetrapeptide carboxypeptidase
slt	Lc	Soluble lytic murein transglycosylase
MltB	Lc, CLas, CLso	Membrane-bound lytic murein transglycosylase B
DacD	Lc, CLas, CLso	DacD D-alanyl-D-alanine carboxypeptidase
OppD	Lc	Oligopeptide transporter; ATP-binding component

Features listed are based on the SEED peptidoglycan recycling subsystem. All sequences in *L. crescens* were aligned to *Ca*. L. solanacearum, and *Ca*. L. asiaticus to confirm SEED annotation. Organisms containing each feature are listed below.

*There are two instances of amiD in the *L. crescens* genome one of which appears to be within the LC2 prophage region.

### Phage

All three sequenced *Liberibacter* species contain two temperate phage regions of the family *Podoviridae* in the order *Caudovirales* [7], [9], [10]. The *L. crescens* prophage regions exhibited very low sequence similarity to each other and to those previously described in CLas and CLso. The prophage regions in *L. crescens* contain proteins of high sequence similarity to integrase, exonuclease, lysozyme, terminase, portal protein, head protein, and other structural proteins common to *Podoviridae* (Table S2). A single phage protein of unknown function is conserved among all three species with a sequence identity greater than 25%.

The very low sequence similarity observed between the integrated prophages of the *L. crescens* and CLas and CLso suggests that the phages were acquired after the lineages of these bacteria diverged. The functions conserved in these prophages are primarily structural in nature which may indicate that all have the potential to re-enter a lytic growth phase.

### Virulence and Host Interaction

#### Effectors

Extra-cytoplasmic proteins produced by CLas and CLso may be interacting with their plant and insect hosts. A previous study predicted 107 potentially secreted proteins encoded by the CLas genome [16]. Homologs to 60 of these proteins were also identified in the *L. crescens* genome. Of those 47 effector proteins not found in *L. crescens*, 16 are also present in CLso. The majority of the potential effectors identified in CLas and CLso are hypothetical with no established function. The phenotypes of many of the 59 effector proteins in common with all *Liberibacter* can be studied in *L. crescens* as this strain is culturable.

#### Indole-3-acetamide Pathway

As with many plant associated bacteria [55], [56], [57], the *L. crescens* genome encodes both proteins necessary for the production of indole-3-acetamide (IAM). These genes, IAM hydrolase and tryptophan monooxygenase, were not found in CLas or CLso. IAM hydroxylase and tryptophan monooxygenase cluster together on the *L. crescens* genome and are most closely related to orthologs in *Pseudomonas syringae* pv. syringae B728a and *Pseudomonas chlororaphis* sbsp. aureofaciens 30–84 respectively. A member of the *Pseudomonas syringae* group was isolated from papaya [58] and the IAM pathway in *L. crescens* may represent a horizontal gene transfer event from a related *Pseudomonas*. Auxin production by *Pseudomonas syringae* is thought to increase its virulence by overriding the plant's natural defenses [59]. However, in *Agrobacteria tumefaciens*, a close relative to *Liberibacter crescens*, auxin production is linked to tumor formation [60]. A dedicated IAM efflux system was not found in any of *Liberibacter* genomes. The auxin efflux carrier (AEC)-family secondary transporter in *Agrobacterium tumefaciens* C58 consists of two proteins Atu0080 and Atu1795, neither of which were found in *L. crescens*. AEC from *P. syringae* was also queried against the *L. crescens* genome with no similar sequences detected. The presence of the IAM pathway in *L. crescens* may indicate a role in plant colonization and potentially pathogenesis.

#### Bacteriocin Production

All three *Liberibacter* genomes possess the Colicin V production pathway. These genes are most closely related to those in *R. leguminosarum* and exhibit the same gene clustering. *L. crescens* and CLso encode the DedE bacteriocin. The *dedE* gene in CLas has been greatly degraded and is unlikely to be functional. The analogous colicin V toxin gene in *R. leguminosarum* was previously shown to have heteroantagonistic activity and was most effective against closely related strains [61].

A putative ABC transporter in *L. crescens* shares a high amino acid sequence similarity of 69.1% with a Colicin V efflux system in *Burkholderia pseudomallei* [gi: 167826023]. This feature is adjacent to an hlyD family secretion gene that is most similar to the *mchE* gene in *Serratia marcescens* Db11 [EMBL: AGE18134], which is associated with the efflux of that organism's microcin peptide. This system is the probable route for colicin v efflux in *L. crescens*. The *L. crescens* bacteriocin may have applications for the control of both CLas and CLso.

## Conclusions

The uncultured plant pathogens *Ca*. L. asiaticus and *Ca*. L. solanacearum have undergone greater genome reduction than their closest cultured relative, *L. crescens*. In the process, CLas and CLso have eliminated many metabolic and regulatory functions that may be needed for growth on artificial medium. The contribution of these biosynthetic deficiencies to the current inability to maintain CLas and CLso in culture is unknown. However, many are likely compensated for through the addition of vitamins and amino acids to culture media.

CLas and CLso have lost an alternate terminal cytochrome, the stringent response, and multiple two component regulatory systems during genome reduction. These losses reduce CLas and CLso's ability to sense and adjust to environmental fluctuations. As an intracellular pathogen, CLas and CLso may not be exposed to drastic environmental changes. However, this diminished flexibility may reduce their ability to effectively transition from the intracellular environment to a free-living state in culture.

CLas and CLso membrane integrity and rate of replacement may be compromised due to the lack of *lpxL* and several enzymes for the recycling of peptidoglycan components. Collectively, these losses may result in a more fragile cell that is highly susceptible to physical damage and environmental stressors that it would not encounter in the intracellular environment but which are difficult to avoid when transferring to artificial growth medium.

Very little has been determined about the mechanism underlying disease development in HLB and psyllid yellows. *L. crescens* has an indole-3-acetamide production pathway not found in CLas and CLso, which may affect its interaction with the plant host. *L. crescens* and CLso also encode a bacteriocin similar to those shown to inhibit the growth of closely related strains in other alphaproteobacteria. The dearth of differences in pathogenicity or virulence genes between *L. crescens* and the pathogens CLas and CLso suggests that *L. crescens* may be a pathogen on an as yet unidentified plant host. Alternatively, hypothetical proteins in CLso and CLas that are absent in *L. crescens* may be playing a role in pathogenicity. Other alternatives include the possibilities that *L. crescens* may serve as a plant growth-promoting symbiont or may exist in plants solely as a non-pathogenic endophyte.

No lone short-coming was found in the CLas and CLso genomes that explains their inability to grow under the same conditions as their closest cultured relative, *Liberibacter crescens*. Rather it seems to be a combination of metabolic, structural, and regulatory processes working in concert to facilitate the independent growth of *L. crescens*. The study of select *L. crescens* knock out mutants is anticipated to expand on this genome comparison and assign additional functional significance to the differences observed herein. Further description and genomic sequencing of *Liberibacter* species will also improve the sensitivity of metabolic modeling, which may be utilized for the development of growth medium as well as the study of genome reduction in the diversification of this important genus.

## Supporting Information

Figure S1 ***Liberibacter crescens*** **growth.** *Liberibacter crescens* was grown in triplicate in liquid BM7 medium at 28°C and 125 rpm. Optical density was measured at 600 nm in a BioTek Synergy HT Microplate reader.
(PDF)

Figure S2 ***Liberibacter crescens*** **cell.** *Liberibacter crescens* has many functions not found in either CLas or CLso. These fall into four broad categories: regulation, central metabolism, transport, and host associations. Additional cellular components not found in CLas or CLso are shown in red. The cytosolic component of the nitrate two-component system is found in CLso (red stripes) while the outermembrane portion of the type 1 secretion system is not found in any of the genomes studied (grey).
(PDF)

Figure S3 **Phenylalanine, Tyrosine, and Tryptophan Biosynthesis.** *Liberibacter crescens* has a complete shikimate pathway which allows for the biosynthesis of three amino acids not produced by *Ca*. Liberibacter asiaticus or *Ca*. Liberibacter solanacearum.
(PDF)

Figure S4 **Pantothenate and CoA Biosynthesis KEGG map.** All three *Liberibacter* species encode the necessary enzymes for the conversion of Pantothenate to Coenzyme A. Synthesis of Pantothenate from pyruvate is less degraded in *L. crescens* however it is still non-functional. This partial pathway in *L. crescens* appears to represent a combination of genome reduction as well as horizontal gene transfer. Ketol-acid reductoisomerase (EC 1.1.1.86) of *L. crescens* is most closely related to the same enzyme in other members of the Rhizobiales; while 3-methyl-2-oxobutanoate hydroymethyltransferase (EC. 2.1.2.11) and pantoate beta-alanine ligase (EC. 6.3.2.1) share 70 and 65 percent sequence homology respectively with members of the Enterobacteriaceae family.
(PDF)

Figure S5 **The Urea Cycle and metabolism of associated amino acids in the** ***Liberibacter*** **genus.** All three *Liberibacter* species studied lacked the arginase enzyme for the production of Urea from Arginine.
(PDF)

Figure S6 **Carbohydrate Metabolism of** ***Liberibacter*** **species.** (A) Glucose-6-phosphate isomerase is not present in *Ca*. L. asiaticus or *Ca*. L. solanacearum. (B) A modified pentose phosphate pathway may be utilized by CLas and CLso to bypass the glucose-6-phosphate that they lack.
(PDF)

Table S1 **Hypothetical genes of unknown function.** Nucleotide sequence comparison of *L*. *crescens*, *Ca*. L. asiaticus, and *Ca*. L. solanacearum identifying conserved hypothetical genes.
(XLSX)

Table S2 ***Liberibacter crescens*** **prophages.** Putative annotation for prophage genes.
(XLSX)
