# Supplementary figures and images for: Comparative Genomics of Cultured and Uncultured Strains Suggests Genes Essential for Free-Living Growth of Liberibacter
Source: PLoS One. 2014 Jan 8;9(1):e84469. doi: 10.1371/journal.pone.0084469 (PMC3885570; doi:10.1371/journal.pone.0084469)

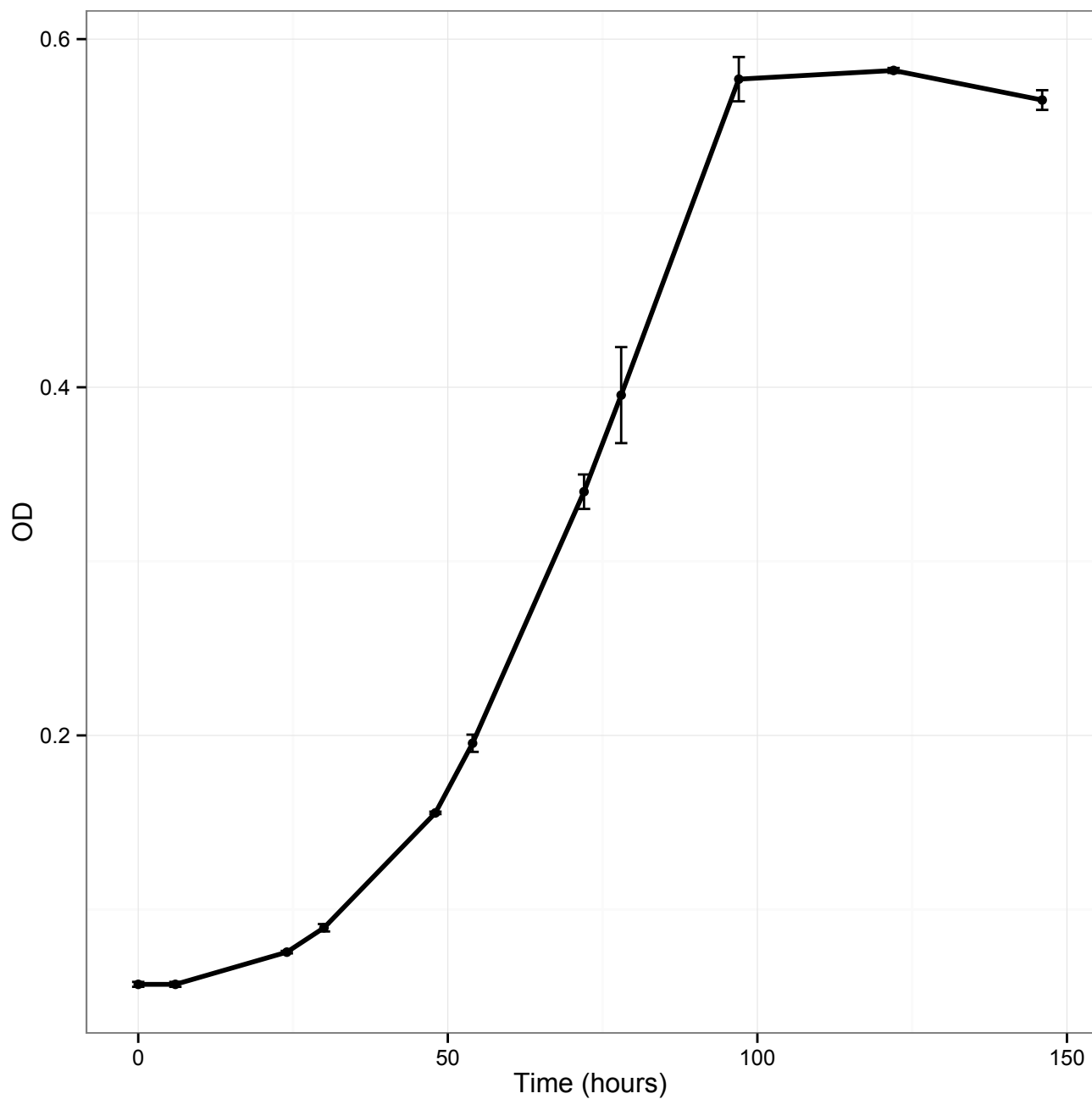

Supplement: Figure S1 — Liberibacter crescens growth. Liberibacter crescens was grown in triplicate in liquid BM7 medium at 28°C and 125 rpm. Optical density was measured at 600 nm in a BioTek Synergy HT Microplate reader. (PDF) [file pone.0084469.s001.pdf]

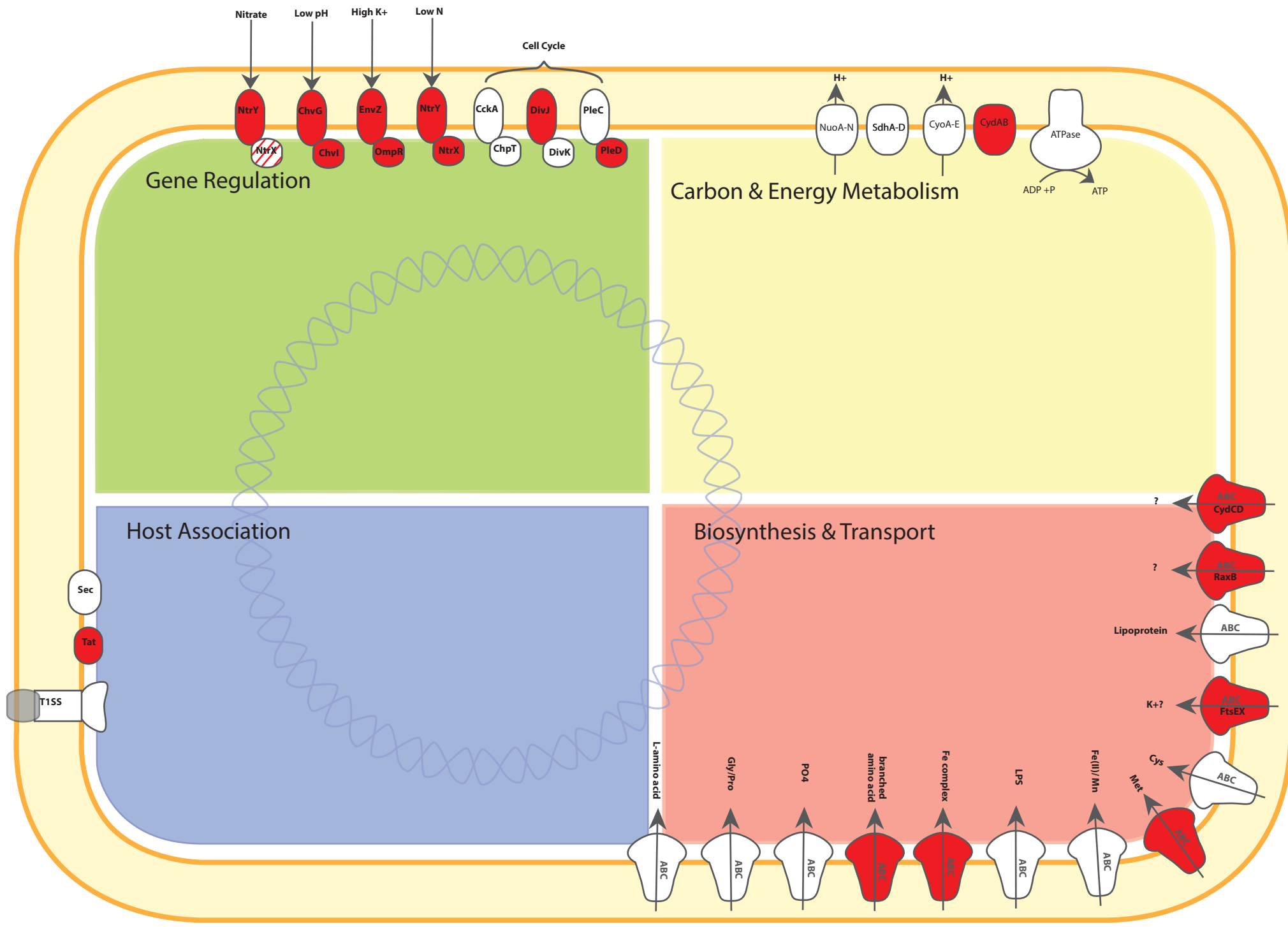

Supplement: Figure S2 — Liberibacter crescens cell. Liberibacter crescens has many functions not found in either CLas or CLso. These fall into four broad categories: regulation, central metabolism, transport, and host associations. Additional cellular components not found in CLas or CLso are shown in red. The cytosolic component of the nitrate two-component system is found in CLso (red stripes) while the outermembrane portion of the type 1 secretion system is not found in any of the genomes studied (grey). (PDF) [file pone.0084469.s002.pdf]

## PANTOTHENATE AND CoA BIOSYNTHESIS

*L. crescens*

Ca. L. asiaticus

Ca. L. solanacearum

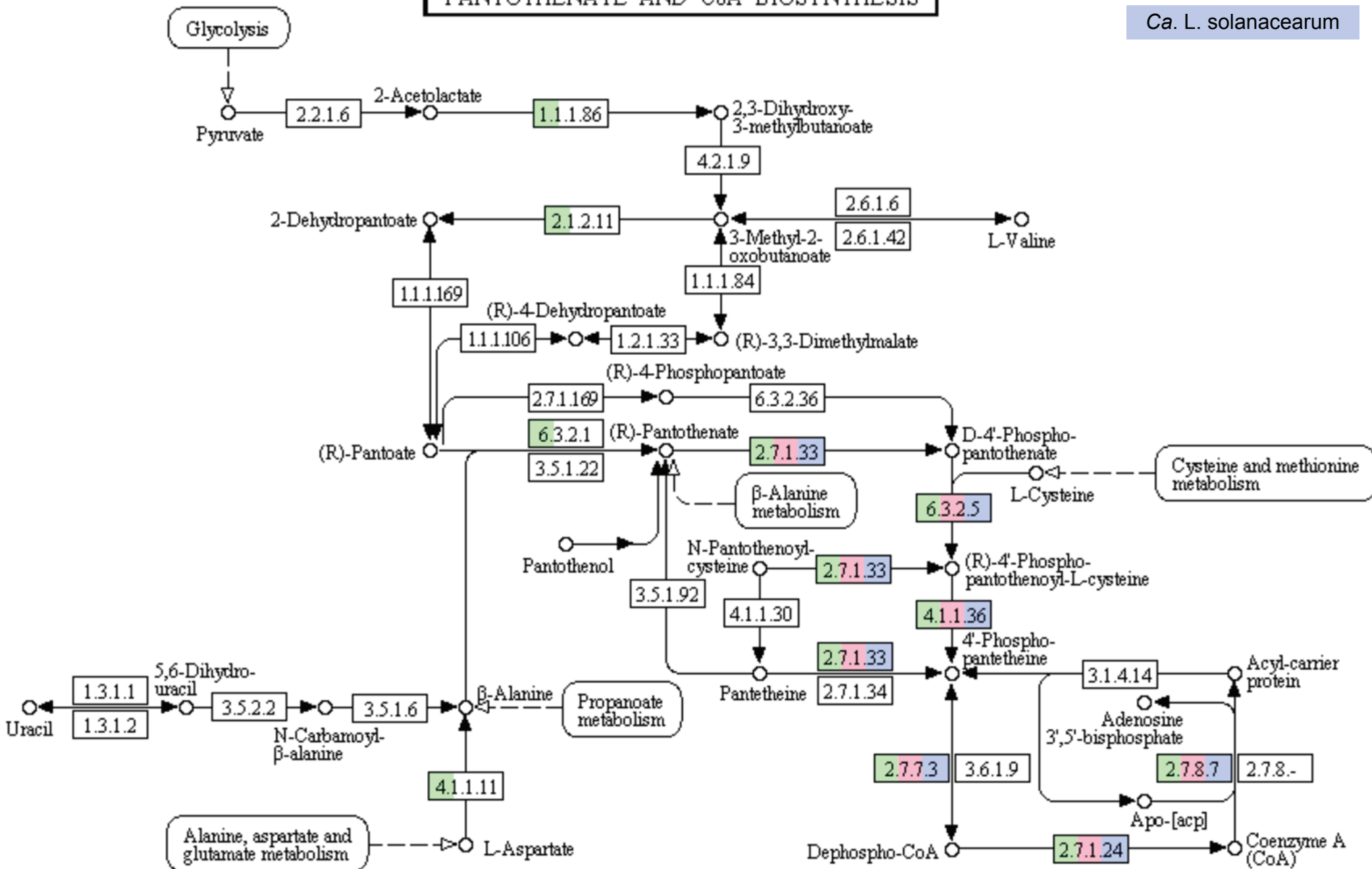

Supplement: Figure S4 — Pantothenate and CoA Biosynthesis KEGG map. All three Liberibacter species encode the necessary enzymes for the conversion of Pantothenate to Coenzyme A. Synthesis of Pantothenate from pyruvate is less degraded in L. crescens however it is still non-functional. This partial pathway in L. crescens appears to represent a combination of genome reduction as well as horizontal gene transfer. Ketol-acid reductoisomerase (EC 1.1.1.86) of L. crescens is most closely related to the same enzyme in other members of the Rhizobiales; while 3-methyl-2-oxobutanoate hydroymethyltransferase (EC. 2.1.2.11) and pantoate beta-alanine ligase (EC. 6.3.2.1) share 70 and 65 percent sequence homology respectively with members of the Enterobacteriaceae family. (PDF) [file pone.0084469.s004.pdf]

# ARGININE AND PROLINE METABOLISM

*L. crescens*

*Ca. L. asiaticus*

*Ca. L. solanacearum*

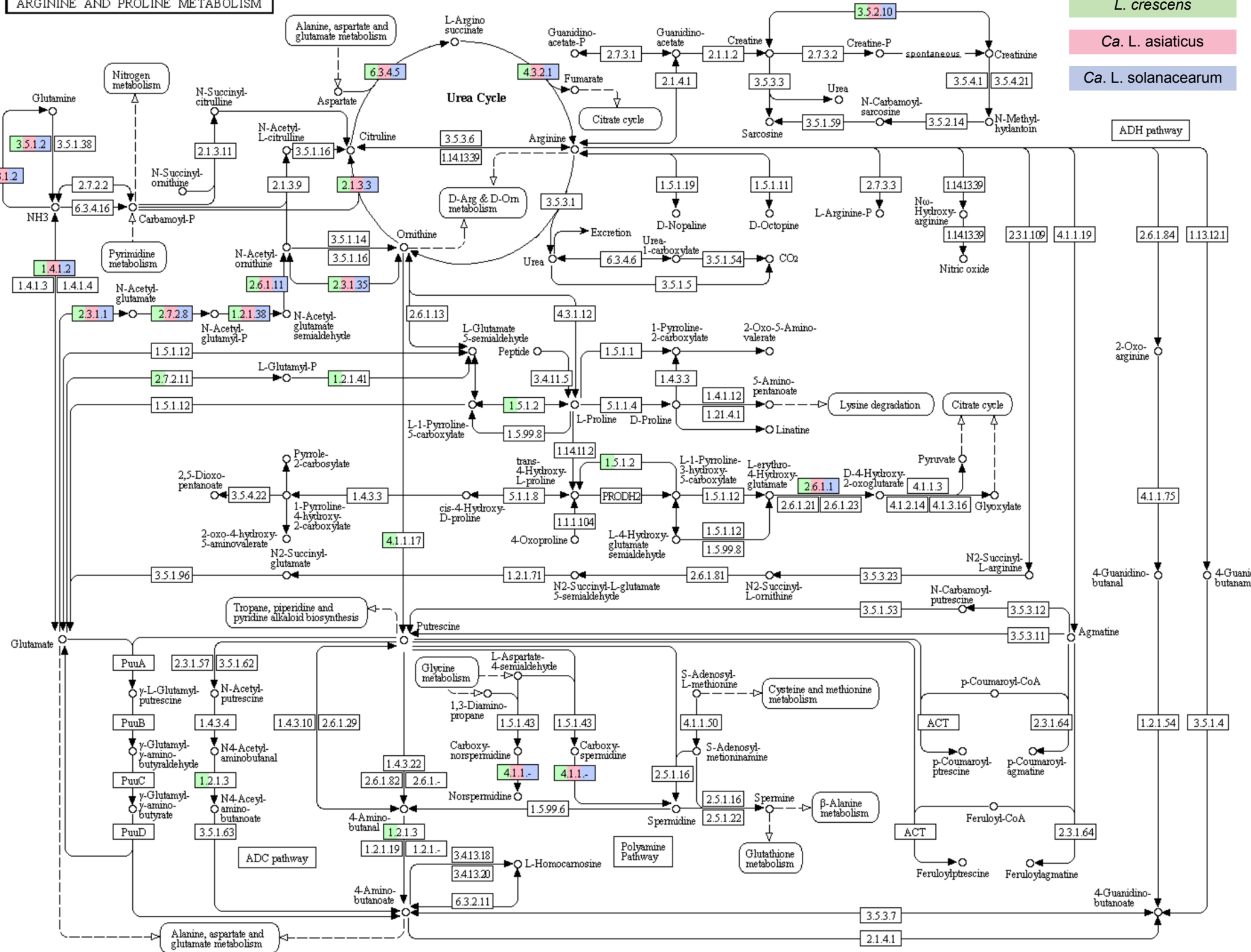

Supplement: Figure S5 — The Urea Cycle and metabolism of associated amino acids in the Liberibacter genus. All three Liberibacter species studied lacked the arginase enzyme for the production of Urea from Arginine. (PDF) [file pone.0084469.s005.pdf]
