# Supplementary material for: Comparative Genomics of Cultured and Uncultured Strains Suggests Genes Essential for Free-Living Growth of Liberibacter
Source: PLoS One. 2014 Jan 8;9(1):e84469. doi: 10.1371/journal.pone.0084469 (PMC3885570; doi:10.1371/journal.pone.0084469)
Supplement: Figure S3 — Phenylalanine, Tyrosine, and Tryptophan Biosynthesis. Liberibacter crescens has a complete shikimate pathway which allows for the biosynthesis of three amino acids not produced by Ca. Liberibacter asiaticus or Ca. Liberibacter solanacearum. (PDF) [file pone.0084469.s003.pdf]

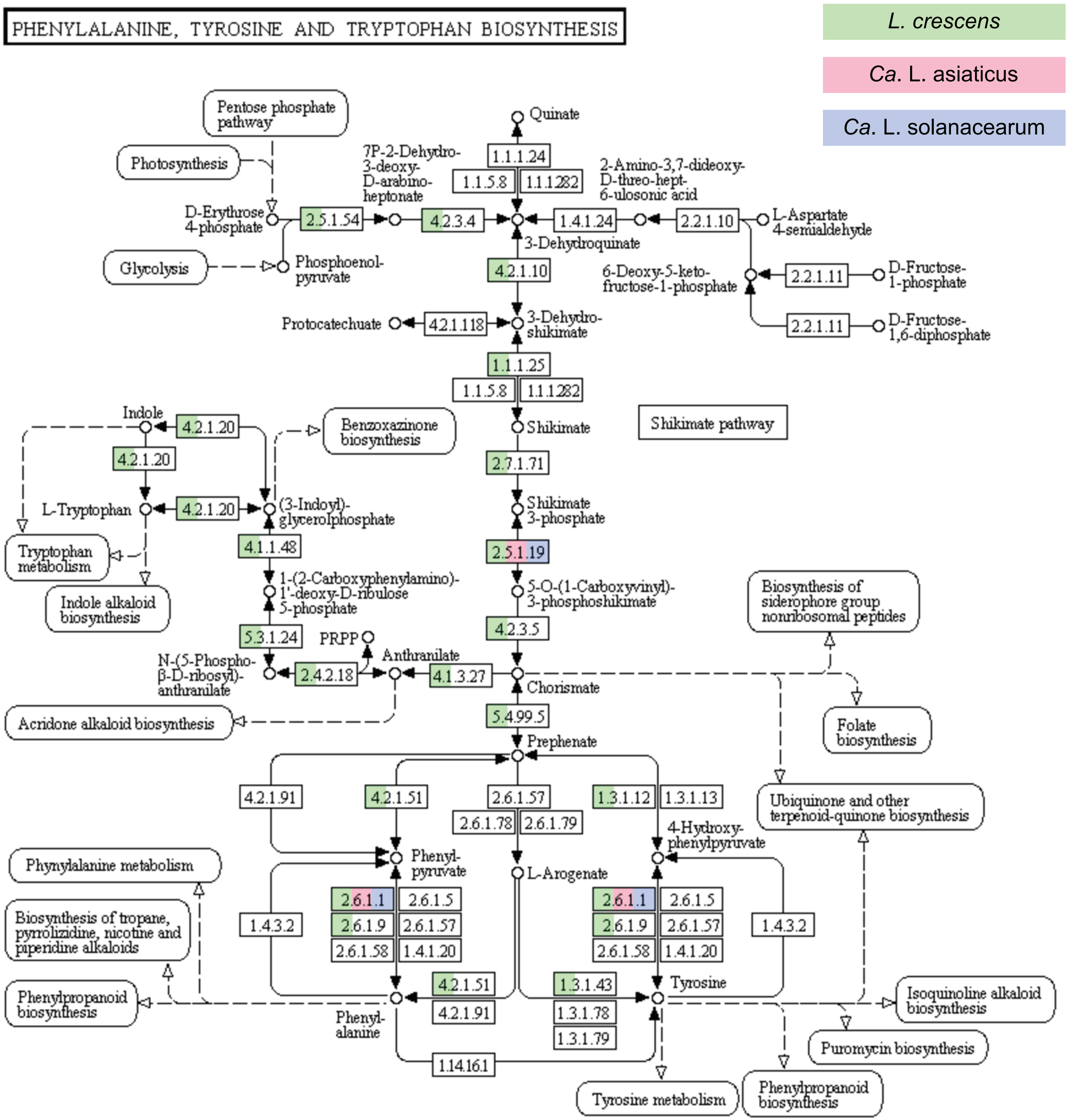
PHENYLALANINE, TYROSINE AND TRYPTOPHAN BIOSYNTHESIS
L. crescens
Ca. L. asiaticus
Ca. L. solanacearum
Pentose phosphate pathway
Photosynthesis
Glycolysis
D-Erythrose 4-phosphate
Phosphoenol-pyruvate
2.5.1.54
7P-2-Dehydro-3-deoxy-D-arabino-heptonate
4.2.3.4
Quinate
1.1.1.24
1.1.5.8
1.1.1.282
3-Dehydroquinate
1.4.1.24
2.2.1.10
2-Amino-3,7-dideoxy-D-threo-hept-6-ulosonic acid
L-Aspartate 4-semialdehyde
6-Deoxy-5-keto-fructose-1-phosphate
2.2.1.11
D-Fructose-1-phosphate
D-Fructose-1,6-diphosphate
4.2.1.10
Protocatechuate
4.2.1.118
3-Dehydro-shikimate
1.1.1.25
Shikimate
Shikimate pathway
2.7.1.71
Shikimate 3-phosphate
2.5.1.19
5-O-(1-Carboxyvinyl)-3-phosphoshikimate
4.2.3.5
Chorismate
Indole
4.2.1.20
Benzoxazinone biosynthesis
L-Tryptophan
(3-Indoyl)-glycerolphosphate
Tryptophan metabolism
Indole alkaloid biosynthesis
4.1.1.48
1-(2-Carboxyphenylamino)-1'-deoxy-D-ribulose 5-phosphate
5.3.1.24
PRPP
Anthranilate
N-(5-Phospho-β-D-ribosyl)-anthranilate
2.4.2.18
4.1.3.27
Acridone alkaloid biosynthesis
Biosynthesis of siderophore group nonribosomal peptides
Folate biosynthesis
5.4.99.5
Prephenate
4.2.1.91
4.2.1.51
2.6.1.57
2.6.1.78
2.6.1.79
1.3.1.12
1.3.1.13
Ubiquinone and other terpenoid-quinone biosynthesis
4-Hydroxy-phenylpyruvate
Phenyl-pyruvate
L-Arogenate
Phynylalanine metabolism
Biosynthesis of tropane, pyrrolizidine, nicotine and piperidine alkaloids
Phenylpropanoid biosynthesis
1.4.3.2
2.6.1.1
2.6.1.5
2.6.1.9
2.6.1.58
1.4.1.20
1.3.1.43
1.3.1.78
1.3.1.79
Tyrosine
Phenyl-alanine
1.14.16.1
Isoquinoline alkaloid biosynthesis
Puromycin biosynthesis
Tyrosine metabolism
Phenylpropanoid biosynthesis
