# Supplementary material for: Comparative Genomics of Cultured and Uncultured Strains Suggests Genes Essential for Free-Living Growth of Liberibacter
Source: PLoS One. 2014 Jan 8;9(1):e84469. doi: 10.1371/journal.pone.0084469 (PMC3885570; doi:10.1371/journal.pone.0084469)
Supplement: Figure S6 — Carbohydrate Metabolism of Liberibacter species. (A) Glucose-6-phosphate isomerase is not present in Ca. L. asiaticus or Ca. L. solanacearum. (B) A modified pentose phosphate pathway may be utilized by CLas and CLso to bypass the glucose-6-phosphate that they lack. (PDF) [file pone.0084469.s006.pdf]

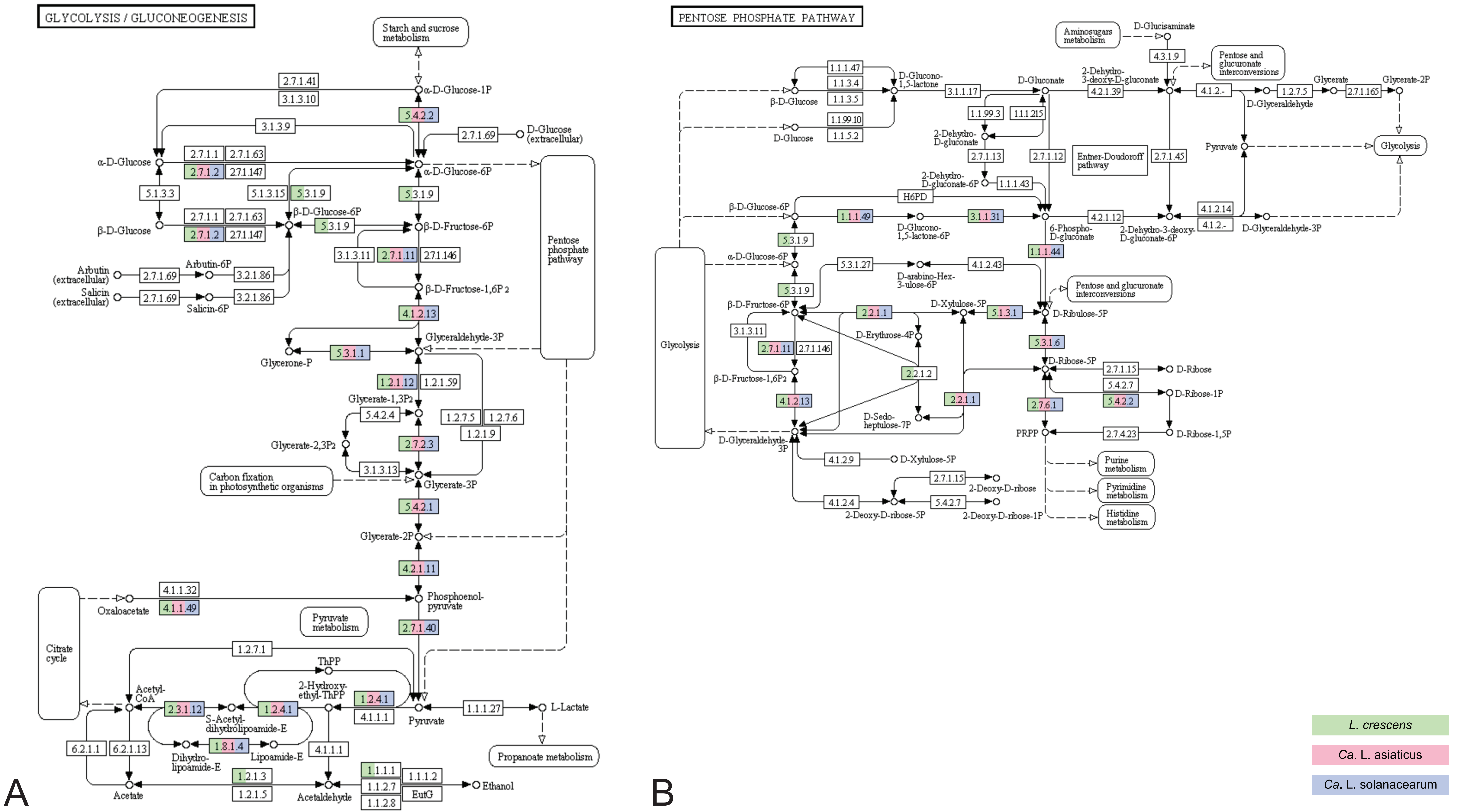
GLYCOLYSIS / GLUCONEOGENESIS
Starch and sucrose metabolism
α-D-Glucose-1P
2.7.1.41
3.1.3.10
5.4.2.2
3.1.3.9
2.7.1.69
D-Glucose (extracellular)
α-D-Glucose
2.7.1.1
2.7.1.63
2.7.1.2
2.7.1.147
α-D-Glucose-6P
5.1.3.3
5.1.3.15
5.3.1.9
β-D-Glucose-6P
β-D-Glucose
β-D-Fructose-6P
Pentose phosphate pathway
3.1.3.11
2.7.1.11
2.7.1.146
Arbutin (extracellular)
Arbutin-6P
3.2.1.86
Salicin (extracellular)
Salicin-6P
β-D-Fructose-1,6P2
4.1.2.13
Glyceraldehyde-3P
5.3.1.1
Glycerone-P
1.2.1.12
1.2.1.59
Glycerate-1,3P2
5.4.2.4
1.2.7.5
1.2.7.6
1.2.1.9
2.7.2.3
Glycerate-2,3P2
3.1.3.13
Carbon fixation in photosynthetic organisms
Glycerate-3P
5.4.2.1
Glycerate-2P
4.2.1.11
4.1.1.32
4.1.1.49
Oxaloacetate
Phosphoenol-pyruvate
Citrate cycle
Pyruvate metabolism
2.7.1.40
1.2.7.1
ThPP
2-Hydroxy-ethyl-ThPP
1.2.4.1
Acetyl-CoA
2.3.1.12
S-Acetyl-dihydrolipoamide-E
1.8.1.4
Dihydro-lipoamide-E
Lipoamide-E
4.1.1.1
Pyruvate
1.1.1.27
L-Lactate
Propanoate metabolism
6.2.1.1
6.2.1.13
1.2.1.3
1.2.1.5
Acetate
Acetaldehyde
1.1.1.1
1.1.1.2
1.1.2.7
1.1.2.8
EutG
Ethanol
A
PENTOSE PHOSPHATE PATHWAY
Aminosugars metabolism
D-Glucisaminate
4.3.1.9
Pentose and glucuronate interconversions
1.1.1.47
1.1.3.4
1.1.3.5
1.1.99.10
1.1.5.2
β-D-Glucose
D-Glucose
D-Glucono-1,5-lactone
3.1.1.17
D-Gluconate
2-Dehydro-3-deoxy-D-gluconate
4.2.1.39
4.1.2.-
D-Glyceraldehyde
1.2.7.5
Glycerate
2.7.1.165
Glycerate-2P
1.1.99.3
1.1.1.215
2-Dehydro-D-gluconate
2.7.1.13
2.7.1.12
Entner-Doudoroff pathway
2.7.1.45
Pyruvate
Glycolysis
2-Dehydro-D-gluconate-6P
1.1.1.43
H6PD
β-D-Glucose-6P
1.1.1.49
D-Glucono-1,5-lactone-6P
3.1.1.31
6-Phospho-D-gluconate
4.2.1.12
2-Dehydro-3-deoxy-D-gluconate-6P
4.1.2.14
4.1.2.-
D-Glyceraldehyde-3P
5.3.1.9
1.1.1.44
α-D-Glucose-6P
5.3.1.27
D-arabino-Hex-3-ulose-6P
4.1.2.43
Pentose and glucuronate interconversions
Glycolysis
β-D-Fructose-6P
2.2.1.1
D-Xylulose-5P
5.1.3.1
D-Ribulose-5P
3.1.3.11
D-Erythrose-4P
2.7.1.11
2.7.1.146
5.3.1.6
D-Ribose-5P
2.2.1.2
2.7.1.15
D-Ribose
β-D-Fructose-1,6P2
5.4.2.7
2.2.1.1
D-Ribose-1P
4.1.2.13
5.4.2.2
2.7.6.1
D-Sedo-heptulose-7P
PRPP
2.7.4.23
D-Ribose-1,5P
D-Glyceraldehyde-3P
4.1.2.9
D-Xylulose-5P
Purine metabolism
2.7.1.15
2-Deoxy-D-ribose
Pyrimidine metabolism
4.1.2.4
5.4.2.7
2-Deoxy-D-ribose-5P
2-Deoxy-D-ribose-1P
Histidine metabolism
B
L. crescens
Ca. L. asiaticus
Ca. L. solanacearum
